# Chondromucosal Nasal Flap With the Transposition Flap of Von Langenbeck as a Good Election for the Total Lower Eyelid Defect Reconstruction in an Old Patient With an Elevated Vision Loss in the Contralateral Eye

**Published:** 2011-06-27

**Authors:** Joaquín Pérez-Guisado, Jesús M. de Haro-Padilla, Luis F. Rioja

**Affiliations:** Service of Plastic, Aesthetic and Reconstructive Surgery, Reina Sofía University Hospital, Córdoba, Spain

## Abstract

**Objective:** The management of lower eyelid reconstruction has a variety of treatment strategies with varying success depending on the patient. We tried to apply the most suitable reconstruction techniques for this particular case. **Methods:** We report a case of a 99-year-old woman, with a vision loss of 70% in the left eye and 40% in the right eye, who underwent basal cell carcinoma resection of the lower right eyelid 3 months before. The margins of resection and the deepest layers were affected. After the histopathology report, we decided to plan a more aggressive treatment with a total resection of the lower right eyelid. We had a case with 3 added difficulties: the old age of the patient, the vision loss in the contralateral eye, and the size of the resection. **Results:** On the basis of our experience and the bibliography reviewed, we decided to use the chondromucosal nasal flap for the posterior lamella reconstruction and the transposition flap of von Langenbeck for the anterior lamella. We found it was a good election for this patient since we achieved good functional, anatomical, and aesthetical results with a one-step operation. **Conclusions:** The chondromucosal nasal flap with the transposition flap of von Langenbeck was a good election for a total lower eyelid defect reconstruction in an old patient with a high vision loss in the contralateral eye.

Skin tumors, especially basal cell carcinomas, are usually the most frequent causes of eyelids defects (80%-90% of incidence for malignant eyelid skin tumors).^1^

There are so many techniques describing the reconstruction of the lower eyelid because it is more affected than the upper eyelid (45% vs 38%^1^ and 80% vs 20%^2^).

In our service, for full-thickness lower eyelid defects, we are used to advancement tarsoconjunctival flap (modified Hughes procedure) technique, because it is a well-known and versatile technique that can be used to reconstruct lower eyelid defects of less than 50% of lid length, as well as defects that fall into the 50% to 75% category.^3^

Large defects affecting more than 50% of the lower lid can be reconstructed with this 2-stage procedure.^4^ Nevertheless, when it concerns to lower eyelid defects of full-thickness measuring greater than 75%, the situation requires a more aggressive approach.^5^

## CLINICAL CASE

The patient is a 99-year-old woman, with a vision loss of 70% in the left eye and 40% in the right eye, who underwent basal cell carcinoma resection of the lower right eyelid 3 months before. The resection had an extension of 0.7 × 0.3 cm^2^, affecting the central margin of the lower eyelid and was treated with direct closure. The hystopathology report of the resection informed solid (nodular) type of basal cell carcinoma characterized by islands of cells with peripheral palisading and disorganized central cells. The margins of resection and the deepest layers were affected.

**Figure 1:**
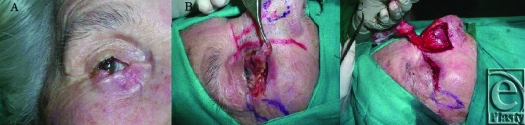
(A) Scar after 3 months of the first resection; (B) Preoperative markings and lower eyelid resection; (C) Chondromucosal nasal flap harvest.

After the hystopathology report, we decided to plan a more aggressive treatment around the scar of the previous resection. The scar was not well defined (Fig 1A), so we thought the best approach would be a total resection of the lower eyelid with a full-thickness resection around the scar that would imply a measurement greater than 75% of the full lower eyelid extension.

We had a case with 3 limitations for applying the advancement tarsoconjunctival flap: the old age of the patient, the elevated vision loss in the contralateral eye, and the high size of the resection. The patient was advanced in years with a severe vision loss in the contralateral eye, so the occlusion of the right eye and the risk of a second step operation would not be the best options. The defect would be too big for applying this technique. Reviewing the bibliography, we thought this patient could be a good candidate for the total lower eyelid reconstruction with the nasal chondromucosal flap, because it does not need eye occlusion and the reconstruction is achieved with one-step operation.^6^

Because of the age of the patient, the operative procedure was performed under local anaesthesia with sedation instead of general anaesthesia. Although this is a large surgical field to perform under local anaesthesia, in our hospital, the final decision about the most suitable anaesthesia depends on the anaesthetist, who considered the local anaesthesia with sedation as the most appropriated method for this patient.

Preoperative markings were done for harvest of the upper lateral cartilage in the nasal chondromucosal flap, which is based on the lateral terminal branch of the dorsal nasal artery, and for transposition flap of von Langenbeck, and then the resection was done (Fig 1B). The piece of resection was 2.8 × 1.9 cm^2^. The flap was designed along the lateral nasal wall and was based on the terminal branch of the dorsal nasal artery, to include the subcutaneous tissues down to the periosteum and the cranial portion of the upper lateral cartilage. The skin was incised for 2.5 cm along the border between the lateral nasal wall and the cheek from the inner canthus to the ala nasi. Then, the subcutaneous tissue was dissected, from lateral to medial, onto a line beyond the midline of the nose. The subcutaneous dissection was extended superiorly to the glabellar area and inferiorly to or beyond the lower margin of the upper lateral cartilages. A portion of the lateral cartilage with 1.2 × 2.1 cm^2^ of size was dissected. The flap was harvested from the pedicle including the portion of the lateral cartilage dissected (Fig 1C).

**Figure 2:**
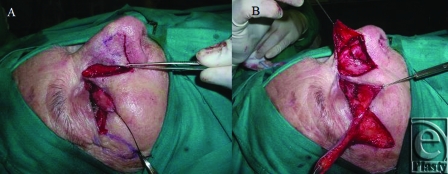
(A) Chondromucosal nasal flap transposition to reconstruct the posterior lamella; (B) Transposition flap of von Langenbeck.

The flap was then transposed to reconstruct the posterior lamella of the lower eyelid (Fig 2A).

With the help of the loupe magnification, flap mucosa was sutured to the conjunctival margin. Transposition flap of von Langenbeck was used for repairing the anterior lamella and correcting ectropion^7^ (Fig 2B), lateral and medial periosteal fixation was performed to minimize the risk of lid malposition. The transposition flap of von Langenbeck consisted in a superiorly based lateral transposition cutaneous flap. Its base was immediately adjacent to the defect, with a length and a width approximately equal to the length and width of the defect. The flap was harvested and transposed into the defect.

The narrow skin bridge created by the extensive lateral nasal incision and the medial extent of the lid resection was reinforced with the stitches from the medial extent of the transposition flap. Wound closure was accomplished by a simple discontinuous subcutaneous pattern (4-0 polyglactin), continuous cutaneous pattern for the transposition flap of von Langenbeck (5-0 nylon), and discontinuous cutaneous pattern for the rest (4-0 nylon). The results, just after (Fig 4A), 3 months after (Fig 4B), and 6 months (Fig 4C) after the surgery, were aesthetically and functionally excellent, and as we can see, with the absence of any malposition, entropion or ectropion. This patient had a good result probably due in part to her aged skin, so maybe the procedure described would be a good alternative just in old people but not in young people.

## DISCUSSION

The management of lower eyelid reconstruction has a variety of treatment strategies with varying success. Advancement tarsoconjunctival flap (modified Hughes procedure), tarsoconjunctival flap (Hewes flap) and posterior lamella grafts (bucal mucosa, nasal chondromucosa, and ear cartilage) are considered as the election techniques for the reconstruction of the posterior lamella. Meanwhile semicircular rotational flap (Tenzel), vertical myocutaneous cheek-lift flap, Tripier flap, rotation cheek flap (Mustardé flap) and temporal forehead flap (Fricke) are for the anterior lamella.^4^

We are going to explain our arguments to rule out the mentioned techniques and why we preferred one procedure to another in this case report.

For the posterior lamella reconstruction, we ruled out the following:

1. Advancement tarsoconjunctival flap (modified Hughes procedure): It is for lower eyelid defects involving greater than 60% of the lid margin and limited to 7 mm in height.^8^ The partial sacrifice of the upper lid, the temporary occlusion of the eye (6-8 weeks), and the height of the defect (6-19 mm) were our arguments to rule out this technique.
2. Tarsoconjunctival flap (Hewes flap): The Hewes flap is indicated for repair of shallow, posterior lamellar, lower lid defects.^4^ The defect was deep so we rule out this procedure.

For the posterior lamell a reconstruction, we agree with Hilko Weerda^7^ considering the chondromucosal nasal graft as the most suitable for the total lower eyelid reconstruction. Since on the contrary of the bucal mucosa graft that provide just mucosa or the ear cartilage that provides just cartilage, the chondromucosal nasal graft^7^ or the chondromucosal nasal flap^6^ provide in one step both for the reconstruction of the mucosa and the tarsal.^6^ We finally decided to use the chondromucosal nasal flap instead of the chondromucosal nasal graft because we considered that the flap could be safer than the graft, although this technique is more complicated.

Concerning to the anterior lamella reconstruction:

1. Semicircular rotational flap (Tenzel): A Tenzel flap is indicated for central or medial defects of the eyelid affecting up to 60% of the eyelid margin, so it was not enough for the size of our defect. Moreover, if this flap is used to repair excessively large defects, a greater amount of unsupported tissue results in the lateral lid, potentially resulting in eyelid malposition including entropion or ectropion.^8^
2. Bipedicle upper eyelid flap (Tripier): The height of the defect that can be repaired is limited to 10 to 15 mm^8^; therefore, it was not appropriate for our reconstruction.
3. Temporal forehead flap (Fricke): Although this transposition flap from the temporal-forehead area was initially described by Fricke for reconstruction of the upper eyelid, it could also be used for lower eyelid reconstruction.^4^ Nevertheless, our experience has taught us that due to the closer distance from the flap to the upper eyelid than the lower eyelid this technique has better results for the upper eyelid.

We found both transposition flap of von Langenbeck and rotation cheek flap (Mustardé flap) as the most appropriated techniques for this case. We finally decided to use transposition flap of von Langenbeck because this technique is less traumatic and better to avoid or even correct a residual ectropion.

We can conclude the chondromucosal nasal flap with the transposition flap of von Langenbeck was a good election for a total lower eyelid defect reconstruction in an old patient with a high vision loss in the contralateral eye. We achieved good functional, anatomical, and aesthetical results with a one-step operation.

## Figures and Tables

**Figure 3:**
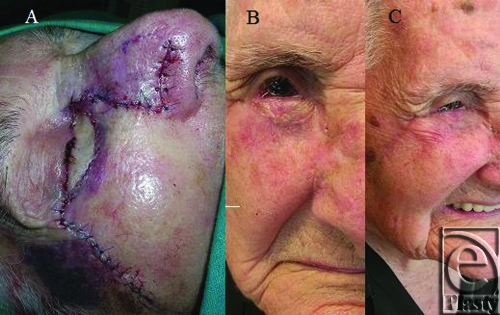
(A) Results just after the surgery; (B) Results 3 months after the surgery; (C) Results 6 months after the surgery.
